# Relationships among cortical activation, cognition, and blood biomarkers in two types of dementia determined using functional near-infrared spectroscopy

**DOI:** 10.3389/fneur.2025.1488420

**Published:** 2025-04-14

**Authors:** Nairong Ruan, Ming Liang, Yuehong Liu, Xi Mei, Chengying Zheng

**Affiliations:** ^1^Department of Psychiatry, Affiliated Kangning Hospital of Ningbo University, Ningbo, Zhejiang, China; ^2^Department of Psychiatry, Ningbo Psychiatric Hospital, Ningbo, Zhejiang, China; ^3^Department of Psychiatry, The Third People's Hospital of Xiangshan County, Ningbo, Zhejiang, China

**Keywords:** cortical activation, Alzheimer's disease, Lewy body dementia, functional near-infrared, biomarker

## Abstract

**Objective:**

The most prevalent types of dementia in older adults are Alzheimer's disease (AD) and Lewy body dementia (LBD), and they have overlapping clinical symptoms. We aimed to define amounts of cortical activation and to identify indicators of brain function to better distinguish between these types of dementia and aid diagnosis using functional near-infrared spectroscopy (fNIRS).

**Methods:**

Oxygenated hemoglobin (HbO) concentrations in the brains of patients with AD and LBD were detected using fNIRS. Brain function was assessed using a verbal fluency task (VFT). Resting-state and task-state cortical activations were investigated to determine differences between AD and LBD. Blood samples were analyzed to identify relevant biomarkers. The clinical and HbO variables were compared between AD and LBD. Functional connectivity at rest and correlations between HbO variables and blood biomarkers were analyzed. The sensitivity and specificity of the parameters for differentiating the dementias were evaluated using areas under the receiver operating characteristic (ROC) curves (AUCs).

**Results:**

This study recruited 28 inpatients with AD and 25 with LBD. Mean HbO concentrations did not significantly differ in the resting state (*p* > 0.05), whereas functional connectivity significantly differed (*t* = −3.449, *p* = 0.001) between the groups. Mean HbO concentrations during the VFT, were significantly lower in the left temporal (*p* = 0.031), right dorsolateral prefrontal (*p* = 0.001), and right temporal (*p* = 0.011) cortices of the AD, than the LBD group. Blood amyloid-β (Aβ)_42_ levels were significantly higher in the AD group (*p* = 0.023), whereas significantly more α-synuclein was expressed in the LBD group (*p* = 0.012). Correlation analysis of cognition-related blood biomarkers with HbO concentrations associated higher plasma Aβ_42_ level with lower HbO concentrations in the right pre-motor and supplementary motor cortex (*r* = −0.378; *p* = 0.005) and higher glial fibrillary acidic protein (GFAP) levels in the lower right pars triangularis (*r* = −0.378; *p* = 0.006) at rest. Levels of the blood biomarker Aβ significantly and negatively correlated with HbO concentrations in the right temporal cortex (*r* = −0.329, *p* = 0.016) during the VFT. The AUC was significantly higher for the combination of multiple fNIRS indicators compared with individual cognitive or blood indicators (AUC = 0.9314).

**Conclusion:**

The characteristics of HbO measured using fNIRS can help distinguish AD from LBD in older adults.

## 1 Introduction

Alzheimer's disease (AD) and Lewy body dementia (LBD) are the two most prevalent neurodegenerative diseases among older individuals (1). Some of their clinical manifestations are similar, such as impaired cognitive dysfunction, including memory, learning, orientation, comprehension, judgment, computation, language, and visuospatial functions; significant reductions in daily living, learning, and working abilities, social interactions, and mental, behavioral, and personality abnormalities, which are often accompanied by other symptoms at specific disease stages (2–5).

The differences between the clinical manifestations of AD and LBD include memory impairment. Paracrine amnesia dominates AD but not LBD, and attention and executive functioning might be more impaired during the early stages of AD. Patients with LBD are characterized by significant changes in cognitive functioning within a single day or over several days, whereas cognitive impairment in AD is usually gradual and fluctuation is not as pronounced as that in LBD. People with LBD often experience visual hallucinations, which are often very detailed and vivid. Patients with AD might also experience hallucinations, but they are not as prevalent or significant as in LBD. Symptoms of Parkinson syndrome, such as bradykinesia, resting tremors, or myasthenia gravis are more prevalent in LBD than in AD. Patients with LBD might have disordered rapid eye movement (REM) sleep behavior characterized by sleep movements and vocalizations that mimic dreams, whereas such manifestations are rare in patients with AD (6–8).

In addition to clinical manifestations, numerous biomarkers have been used to assist in the differential diagnosis of AD and LBD (9). Such biomarkers are detected by cerebrospinal fluid (CSF) analysis, blood-based assays, and noninvasive neuroimaging techniques such as magnetic resonance imaging (MRI), positron emission tomography (PET) imaging, and electroencephalography (EEG) (10–12). Functional near-infrared spectroscopy (fNIRS) has recently been applied to investigate brain activity in patients with cognitive impairment (13). Compared with MRI, fNIRS is portable comfortable, less noisy, inexpensive, insensitive to motion, and has high temporal resolution.

The fNIRS-based verbal fluency task (VFT) has been applied to study impaired phonemic fluency, which is associated with a higher Lewy body and tangle burden in the frontal, temporal, and limbic regions, and impaired semantic fluency, which is associated with greater limbic pathology. Although neurofibrillary tangles tend to be more dense in several regions in patients with impaired verbal fluency, increased Lewy body density is generally associated with verbal fluency deficits. Compared with patients with AD, patients with LBD have better verbal learning performance but worse verbal forgetting and recency effects (14, 15). These specific measures of verbal memory can be used as cognitive markers to differentiate these conditions. Blood biomarker levels of total tau are also higher in patients with AD than in those with LBD.

fNIRS has been used to investigate and distinguish between different types of dementia and healthy older individuals or those with mild cognitive impairment (16, 17). However, distinguishing between AD and DLB has not been investigated in detail, and more investigation is needed. This study used fNIRS to compare prefrontal, temporal, and partial parietal cortex activity in patients with AD and LBD during the VFT, and determined correlations among blood biomarkers, oxygenated hemoglobin (HbO) concentrations, and cortical activation. Our results showed that fNIRS combined with blood parameters significantly increased the sensitivity of a differential diagnosis.

## 2 Methods

### 2.1 Participants

We recruited 28 and 25 patients with AD and LBD, respectively, from the Geriatric Psychiatric Center of the Affiliated Kangning Hospital of Ningbo University between October 2023 and July 2024. Patients with AD or LBD were diagnosed using the Diagnostic and Statistical Manual of Mental Disorders, Fifth Edition. All participants met the following inclusion and exclusion criteria: diagnosis by two research psychiatrists, provided written informed consent, and disease course > 6 months. Caliper matching was implemented to match AD and LBD patients by age, sex, and educational attainment, permitting a specified tolerance range.

### 2.2 Neuropsychiatric evaluations

Memory, language, and other types of cognitive impairment in the patients were evaluated using the Mini-Mental State Examination (MMSE) scale and Alzheimer's Disease Assessment Scale-cognitive subscale (ADAScog) (18). The MMSE is a 30-point tool used to screen cognitive impairment. Severe dementia is often indicated by MMSE scores <11 (19). The ADAScog 70-point scale (higher scores indicate worse impairment) is primarily used to track cognitive decline. Scores > 49 indicate severe dementia.

### 2.3 Study protocols

**Figure 1:**
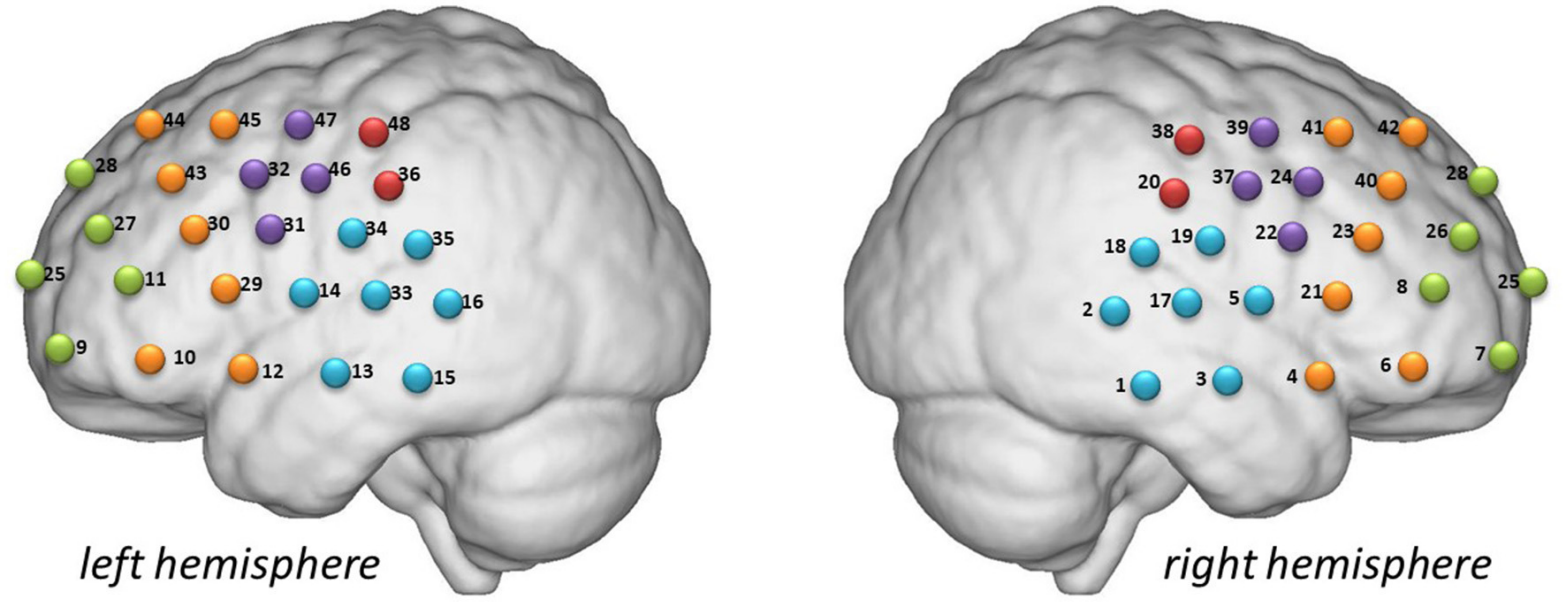
Detection locations. Green: frontopolar area (channels 7, 8, 9, 11, 25, 26, 27, and 28). Purple: left and right pars triangularis Broca areas (right, 22, 24, 37, and 39; left, 31, 32, 46, and 47). Red: left and right pre-motor and supplementary motor cortexes (right, 20 and 38; left, 36 and 48). Blue: left and right temporal cortexes (right, 1, 2, 3, 5, 17, 18, and 19; left, 13, 14, 15, 16, 33, 34, and 35). Yellow: left and right dorsolateral prefrontal cortexes (right, 4, 6, 21, 23, 40, 41, and 42; left 10, 12, 29, 30, 43, 44, and 45).

Figure 1 shows the locations investigated in this study. The participants sat comfortably in a quiet room and remained calm during the 600 s detection period associated with the resting-state task. The VFT consisted of 30 and 70 s pre- and post-task baselines, respectively, during which the participants were required to repeat counting from one to five. During the task period, the participants were required to construct as many phrases as possible using three commonly used characters, such as “bai” (white), “xiao” (small), and “yun” (cloud). The three characters were changed every 20 s during the task to reduce the time during which the patients were silent.

### 2.4 fNIRS measurement and processing

The patients sat in a comfortable chair and adopted a relaxed posture. The experiment then started, and the patients moved minimally throughout the procedure. The test environment was rigorously controlled to ensure acoustic isolation, with all external auditory disturbances eliminated during data acquisition. The experimental procedure began with the acquisition of resting-state HbO data, followed by the VFT data.

We measured hemoglobin concentrations using a NirScan-6000A multichannel fNIRS brain imaging device (Danyang Huichuang Medical Equipment Co., Ltd., Huichuang, China). The sampling frequency was 11 Hz, and the wavelengths were 730, 808, and 850 nm, with 730 and 850 nm as the major and isotopic wavelengths for corrections, respectively, as described. We used the FPz channel (10/20 international system) as the center of the middle probe. Thirty-one source-detector (SD) probes (15 sources and 16 detectors) with a fixed 3-cm inter-probe distance were placed to cover the bilateral PFC and temporal cortices of the participants. A total of 48 NIRS channels were established.

The NIRS data were analyzed using NirSpark package (V1.7.5, Huichuang, China). The data were preprocessed as follows: motion artifacts were corrected using moving SD and cubic spline interpolation. Physiological noise was removed using a band pass filter with cut-off frequencies of 0.01–0.20 Hz. Optical densities were converted into altered HbO and deoxygenated hemoglobin concentrations using the modified Beer–Lambert law. Instead of deoxyhemoglobin (HbR), we used HbO as our primary indicator in the following analysis because of its high signal-to-noise ratio.

Based on the waveforms of individuals in all 48 channels, the average waveforms of HbO changes in each channel for all patients in four groups were obtained by linear fitting all baseline data. We minimized cutaneous blood flow signal artifacts by implementing advanced computational algorithms and an optimized multi-probe configuration design. The synergistic integration of these technological enhancements reduced hemodynamic interference while maintaining measurement fidelity.

For cerebral activation analysis during the VFT, a 70-s dataset was initially extracted (comprising 10 s of counting baseline and 60 s of verbal fluency task), with the 10-s counting period serving as the baseline to calculate the average HbO concentration during the 60-s VFT period. The slope of HbO concentration during VFT execution was defined as the ascending rate of HbO within the initial 5 s following verbal initiation. Based on Brodmann's parcellation scheme, the cerebral cortex was partitioned into 9 regions of interest (ROIs), where the HbO concentration for each ROI was determined by averaging HbO values across all channels within that specific region. Regarding brain network analysis, the Pearson correlation coefficients of HbO concentration variations among 48 channels during resting-state measurements were employed as quantitative indicators of functional connectivity strength.

### 2.5 Blood collection and plasma processing

Before breakfast, whole blood samples (5 mL) were obtained by phlebotomists into procoagulant tubes using a winged blood collection set, then immediately separated at 1,400 rpm for 10 min using a BY-600A type medical centrifuge (Beijing Baiyang Medical Devices Co., Beijing, China). The samples were processed within 30 min of collection and immediately frozen at −80°C. Blood samples were thawed immediately before analysis.

Serum amyloid-β (Aβ), Aβ_40_, Aβ_42_, total tau, phosphorylated tau (p-tau), p-tau181, α-synuclein (α-syn), neurofilament light (NFL), and glial fibrillary acidic protein (GFAP) levels were estimated using enzyme-linked immunosorbent assay (ELISA) kits (Shanghai Yuanye BioTechnology Co., China) as described by the manufacturer. Absorbance was measured at 450 nm using a Sunrise-basic enzyme labeling instrument (Tecan Co., Männedorf, Switzerland) with a reference wavelength of 690 nm. These measurements were transformed into concentrations by comparing the optical density of the samples with standard curves.

### 2.6 Statistical analysis

Data are presented as means ± standard deviation (SD). Demographic and clinical variables as well as HbO changes to identify differences in cortical activation were compared between patients with AD and LBD using Student *t*-tests. Functional connectivity was analyzed using Pearson correlation coefficients between the time series of each channel-to-channel pair. Correlations between HbO variables and blood biomarkers were analyzed using Pearson correlations. Values with *p* < 0.05 were considered statistically significant. All data were analyzed using IBM SPSS Statistics for Windows, version 19.0 (IBM Corp., Armonk, NY, USA).

## 3 Results

### 3.1 Demographic and clinical data

**Figure 2:**
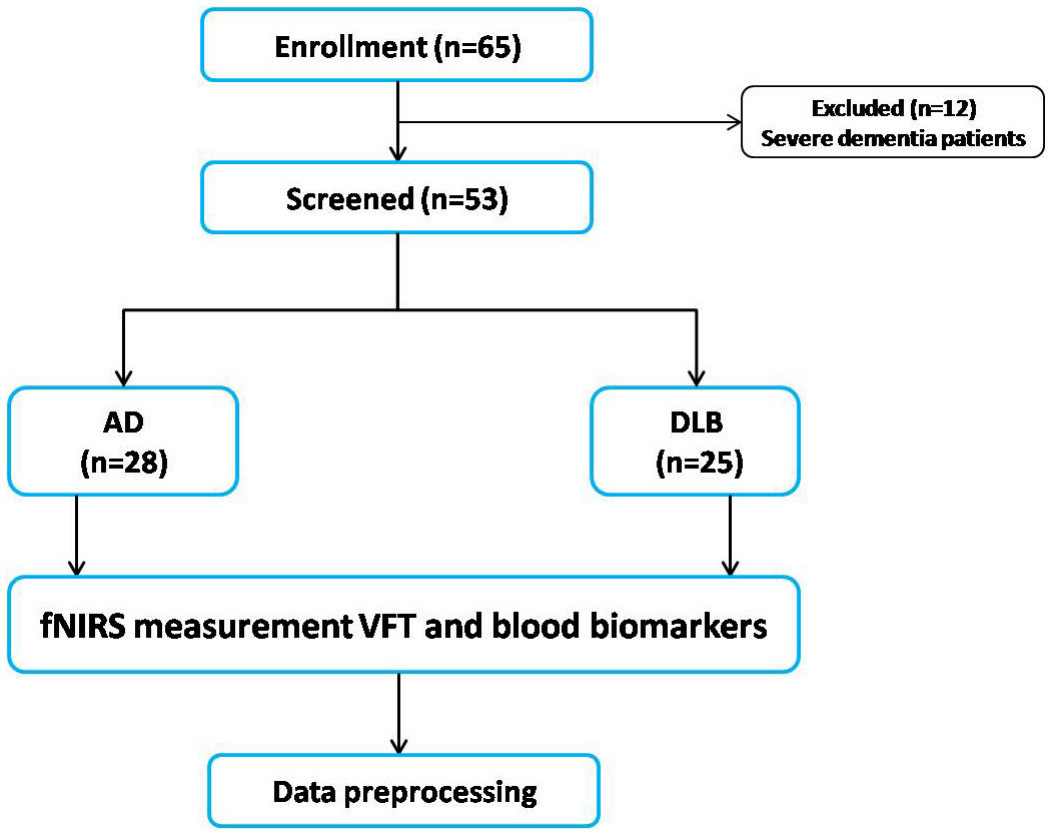
Flowchart of study participant enrollment and measurement protocols. AD, Alzheimer's disease; fNIRS, functional near-infrared spectroscopy; LBD, Lewy body disease; VFT, verbal fluency task.

The flowchart in Figure 2 shows the participant recruitment and measurement protocols. Twelve of 65 initially selected patients could not complete the VFT because of severe dementia. Therefore, we analyzed data from 53 patients. Table 1 shows the demographic characteristics of the participants. The variables of mean age (AD group: range from 62 to 87; DLB group: range from 62 to 84), sex ratio, and education did not significantly differ between the groups (*p* > 0.05). The mean total scores of neuropsychiatric scales in participants with AD were significantly lower for the MMSE (17.50 ± 4.18 vs. 21.20 ± 4.60, *p* = 0.003; AD group: range from 12 to 27; DLB group: range from 13 to 29) and higher for the ADAScog (34.42 ± 14.02 vs. 25.85 ± 16.60, *p* = 0.047; AD group: range from 13.5 to 61.33; DLB group: range from 6.83 to 59.13) compared with those of participants with LBD.

**Table 1:** Participant demographic and clinical data.

**Variable**	**AD (*n* = 28)**	**LBD (*n* = 25)**	***t*/χ^2^**	***p***
Age (years)	74.46 ± 7.22	72.24 ± 6.04	1.208	0.233
Sex (m/f)	8/20	9/16	0.569	0.572
Education (years)	6.21 ± 3.17	6.36 ± 2.27	−0.190	0.850
MMSE score	17.50 ± 4.18	21.20 ± 4.60	−3.069	0.003
ADAScog score	34.42 ± 14.02	25.85 ± 16.60	2.035	0.047

Data are shown as means ± SD.

AD, Alzheimer's disease; ADAScog, Alzheimer's Disease Assessment Scale-cognitive subscale; LBD, Lewy body dementia; MMSE, Mini-Mental State Examination; SD, standard deviation.

### 3.2 Differences in resting-state HbO concentrations between AD and LBD

**Figure 3:**
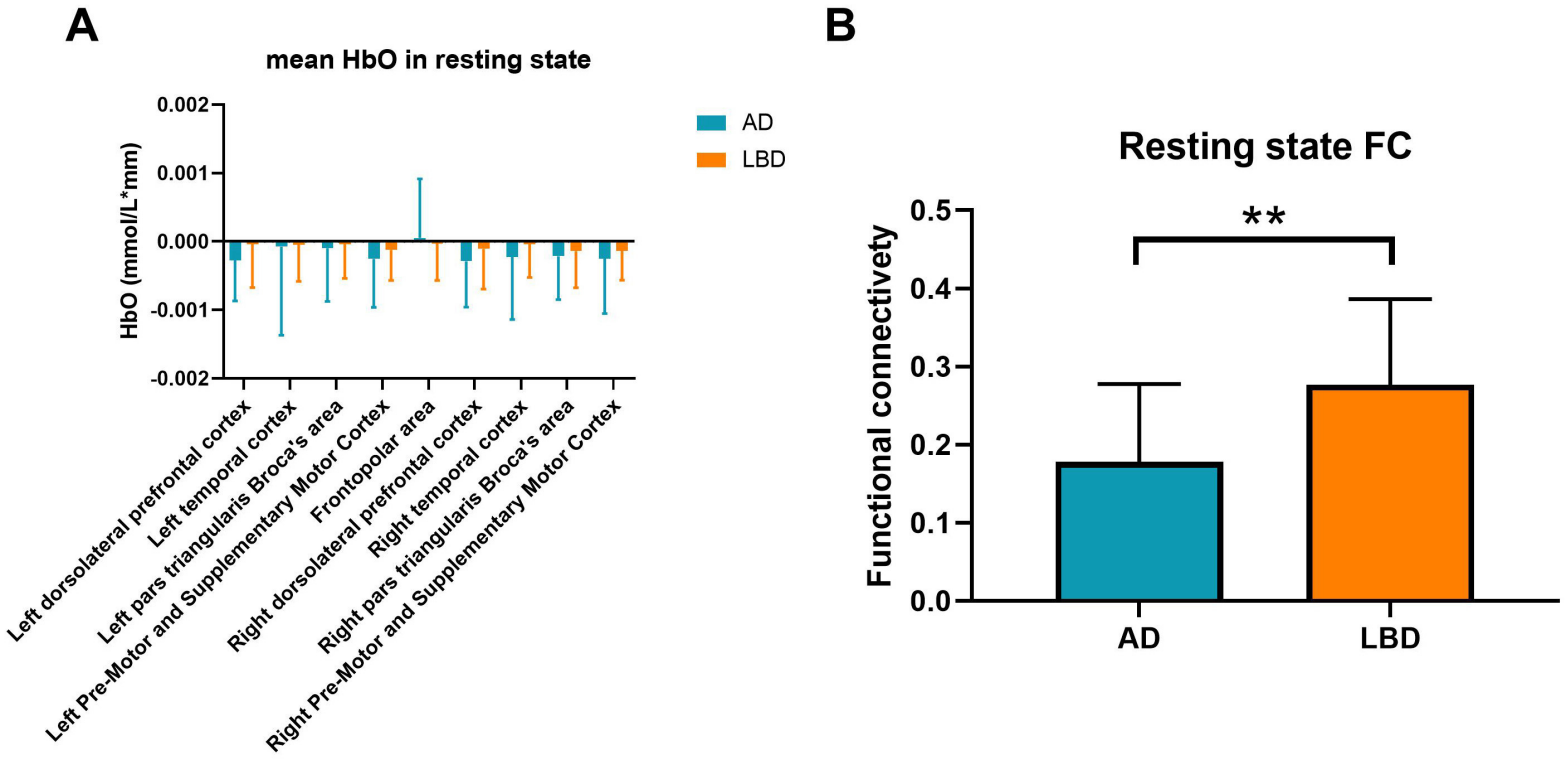
Mean resting-state HbO concentrations **(A)** and functional connectivity **(B)** in patients with AD and LBD. Blue and orange bars: AD and LBD, respectively. AD, Alzheimer's disease; HbO, oxygenated hemoglobin; LBD, Lewy body disease; HbO, oxygenated hemoglobin; FC, functional connectivity. **p < 0.01.

Figure 3A shows that mean resting-state HbO concentrations across the brain regions did not significantly differ between patients with AD and LBD. However, mean resting-state functional connectivity values of the whole brain significantly differed between the AD and LBD groups (0.18 ± 0.09 vs. 0.27 ± 0.11, *t* = −3.449, *p* = 0.001; Figure 3B).

### 3.3 Differences in HbO concentrations during VFT

**Figure 4:**
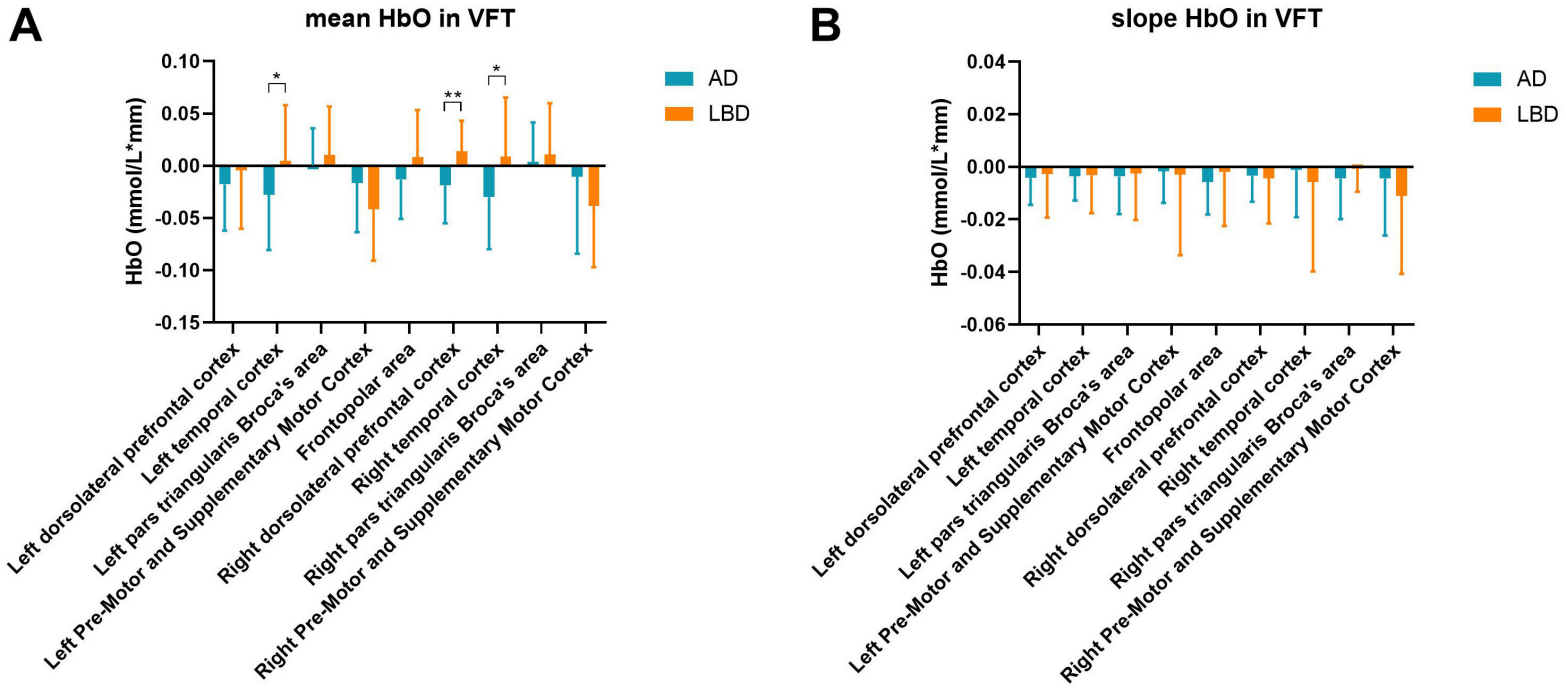
Mean HbO **(A)** and slope of HbO concentrations **(B)** in various brain regions during the VFT. AD, Alzheimer's disease; HbO, oxygenated hemoglobin; LBD, Lewy body disease; VFT, verbal fluency task. *p < 0.05; **p < 0.01.

We investigated mean and slope HbO concentrations in different cortical regions in the patients during the VFT (Figures 4A, B). Compared with the LBD group, the mean HbO concentrations were significantly lower in the AD group in the left temporal cortex (*p* = 0.031), right dorsolateral prefrontal cortex (*p* = 0.001), and right temporal cortex (*p* = 0.011). The slopes of HbO concentrations did not significantly differ between the groups.

### 3.4 Blood biomarker levels differ between the groups

**Figure 5:**
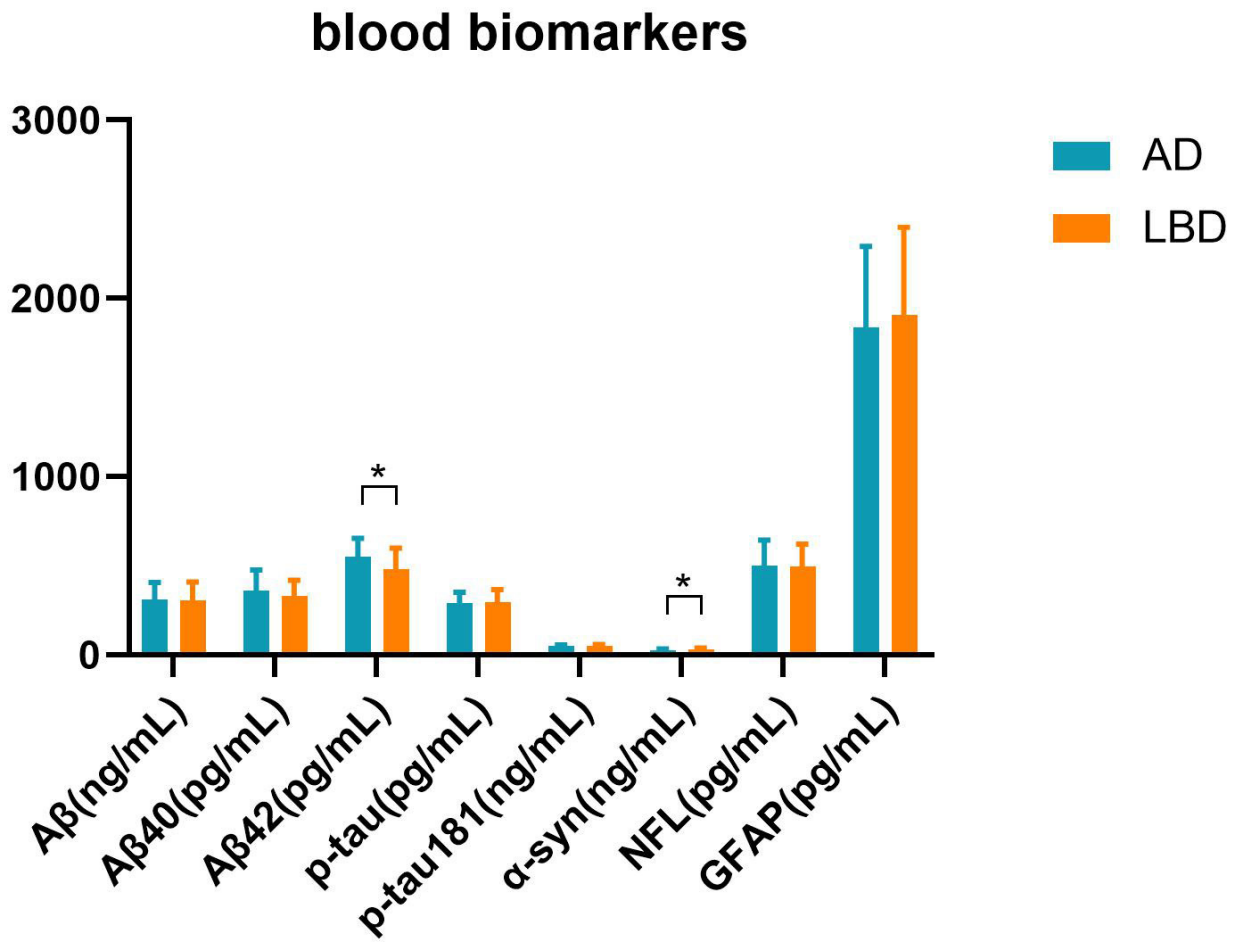
Blood parameters in the AD and LBD groups. We measured cognition-related blood biomarkers: total amyloid-β (Aβ), Aβ_40_, Aβ_42_, phosphorylated tau (p-tau), phosphorylated tau181 (p-tau181), α-synuclein (α-syn), neurofilament light (NFL), glial fibrillary acidic protein (GFAP). *p < 0.05.

We measured levels of Aβ, Aβ_40_, Aβ_42_, p-tau, p-tau181, α-syn, NFL, and GFAP that are indicators of cognition in blood (Figure 5). The value of Aβ_42_ was significantly higher in the AD group than in the LBD group (*t* = 2.336, *p* = 0.023), while α-syn expression was significantly higher in the LBD group than in the AD group (*t* = −2.602, *p* = 0.012).

### 3.5 Correlations between HbO concentrations and blood biomarkers

**Figure 6:**
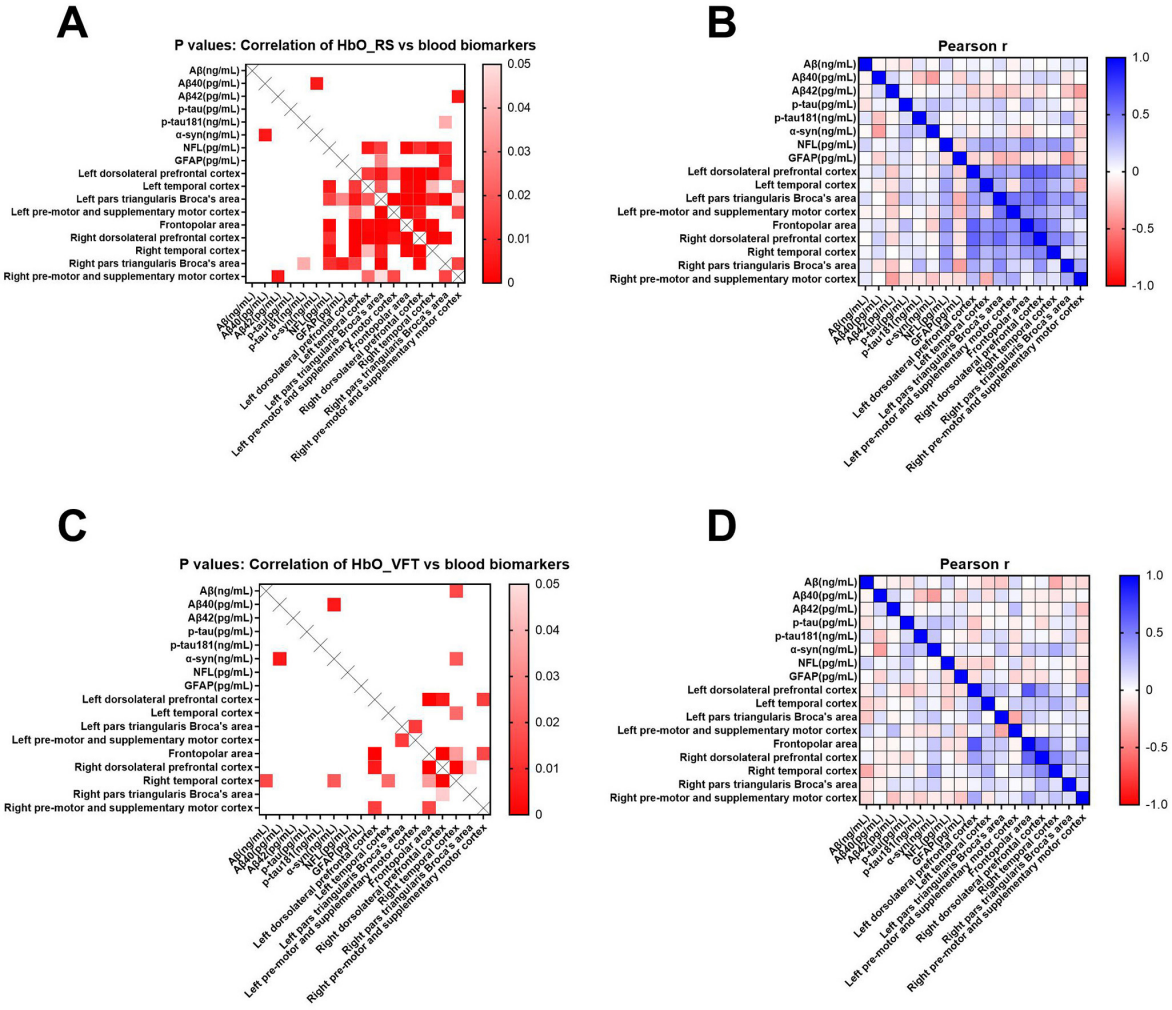
Pearson correlation analyses of cognition-related blood biomarkers with mean resting-state HbO concentrations in brain regions of interest **(A, B)** and VFT **(C, D)**. Blood parameters comprised total amyloid-β (Aβ), Aβ_40_, Aβ_42_, phosphorylated tau (p-tau), phosphorylated tau181 (p-tau181), α-synuclein (α-syn), neurofilament light (NFL), and glial fibrillary acidic protein (GFAP). Scale bars: Pearson coefficient *r* and *p*-values, respectively.

Figure 6 shows correlations between blood biomarkers and mean resting-state HbO concentrations in various brain regions (Figures 6A, B) during VFT (Figures 6C, D). Lower HbO concentrations in the resting state were associated with elevated plasma Aβ_42_ in the right pre-motor and supplementary motor cortices (*r* = −0.378; *p* = 0.005) and elevated GFAP in the lower right pars triangularis (*r* = −0.378; *p* = 0.006) (Figures 6A, B). Resting-state HbO concentrations were associated positively with p-tau181 and NFL in the frontopolar area, right dorsolateral prefrontal and right temporal cortices, and right pars triangularis (p-tau181 *r* = 0.285, *p* = 0.039; NFL *r* = 0.426, *p* = 0.001; *r* = 0.347, *p* = 0.011; *r* = 0.402, *p* = 0.003; *r* = 0.349, *p* = 0.010, respectively). During the VFT, Aβ levels correlated significantly and negatively with HbO concentrations in the right temporal cortex (*r* = −0.329, *p* = 0.016).

### 3.6 Analysis of combined HbO concentrations and blood biomarkers (ROC curves)

**Figure 7:**
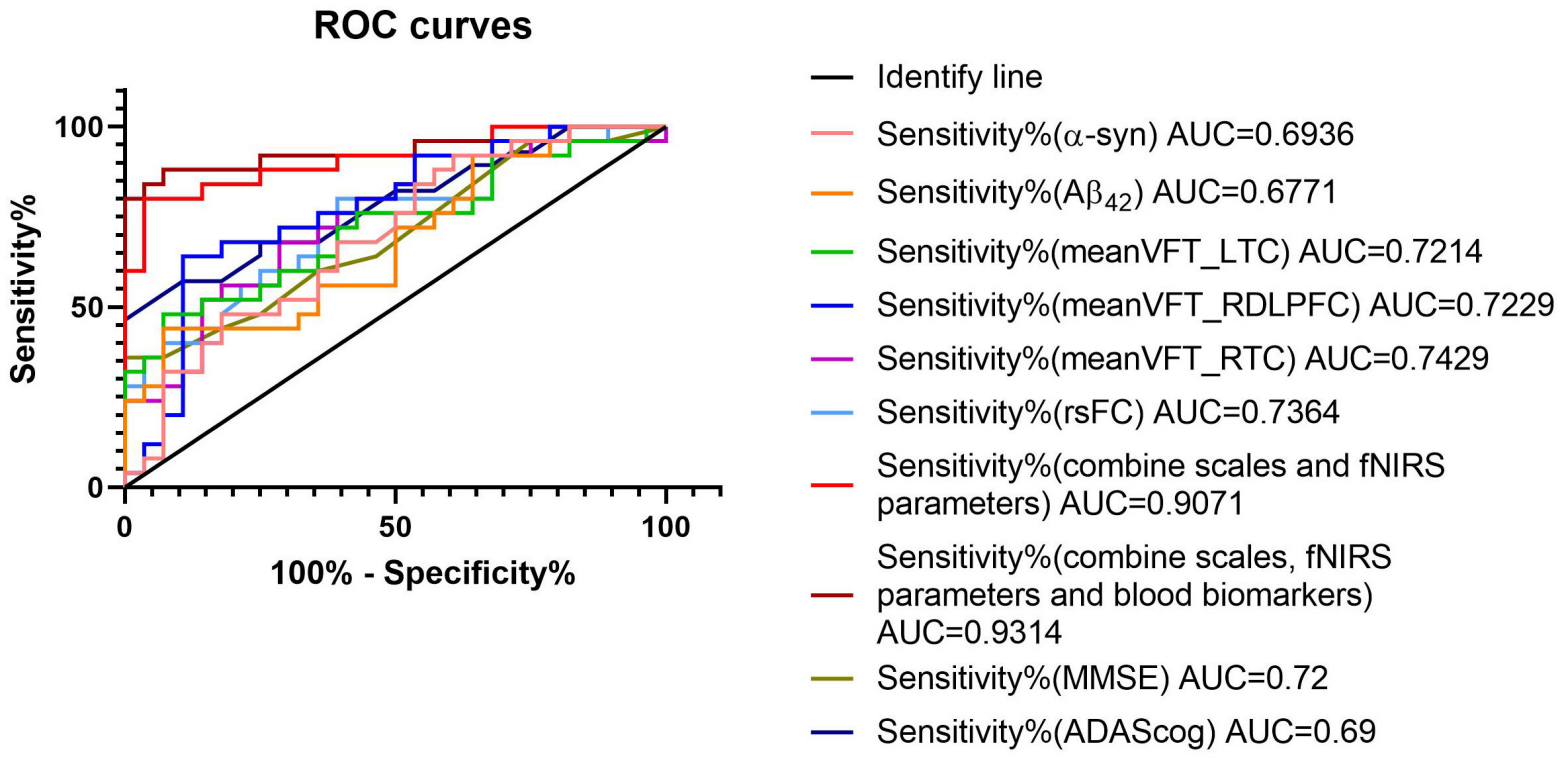
ROC curves of diagnostic parameters. The combination of fNIRS and blood parameters improved sensitivity and specificity. The fNIRS indicators included mean HbO concentrations of left temporal cortex (meanVFT_LTC), right dorsolateral prefrontal cortex (meanVFT_RDLPFC), and right temporal cortex (meanVFT_RTC) during VFT. The blood indicators included amyloid-β (Aβ)_42_ and α-synuclein (α-syn). fNIRS, functional near-infrared spectroscopy; ROC, receiver operating characteristic.

The mean ages of the patients were matched between the AD and LBD groups. The accuracy of dementia diagnoses was enhanced using fNIRS parameters. The AUCs were 0.69 and 0.72 for MMSE and ADAScog, respectively. Figure 7 shows that the AUC was significantly higher after combining multiple fNIRS indicators compared with individual cognitive or blood indicators. The fNIRS indicators included HbO concentrations in the left temporal, right dorsolateral prefrontal, and right temporal cortexes during the VFT. The blood indicators included Aβ_42_ and α-syn levels. The AUC for these combined parameters was 0.9314.

## 4 Discussion

Our findings showed that cortical activation measured using fNIRS during the VFT and blood biomarkers differed between patients with AD and LBD. Combining fNIRS findings and blood biomarkers significantly improved the sensitivity and specificity of differential diagnosis compared with either neuropsychiatric scales or clinical manifestations.

We assessed HbO concentrations reflected by fNIRS under resting state and during VFT. Mean HbO concentrations across the brain regions did not significantly differ between the patients at rest, whereas functional connectivity was stronger for those with LBD than AD. This result is consistent with the findings of an MRI study of the nucleus basalis of Meynert (NBM) that assessed functional connectivity in AD and LBD (20). During the VFT, the mean HbO concentrations were significantly lower in the right dorsolateral prefrontal cortex and left and right temporal cortices in the AD group compared with that of those in the LBD group. The semantic VFT can be used to distinguish between AD, LBD, and behavioral variants of frontotemporal dementia (14). Thus, these differences can be used as neuroimaging biomarkers to distinguish these types of dementia.

The results of investigations into AD pathology and LBD using plasma biomarkers suggested that abnormal levels of plasma NFL and GFAP are associated with increased amyloid levels (21, 22). Other blood biomarkers, including p-tau181, can accurately detect abnormalities in patients with LBD (5). We also investigated correlations between levels of the blood biomarkers amyloid and tau NFL, GFAP, and alpha-syn and HbO concentrations. The LBD group showed significantly higher α-syn expression and lower Aβ_42_ than did those in the AD group. In both groups, higher plasma Aβ_42_ levels were associated with lower HbO concentrations in the right pre-motor and supplementary motor cortices. The strength of correlations was lower in various brain regions during the VFT than while resting. This suggested that dementia confers greater effects on interactions between brain regions during tasks.

Various modalities have been proposed to distinguish AD from LBD (23, 24). Here, we investigated the ability of fNIRS combined with blood biomarkers based on AUC to differentiate LBD from AD. Impaired phonemic fluency in LBD is associated with a higher Lewy body and tangle burden in the frontal, temporal, and limbic regions (25). Patients with AD and white matter pathology in both frontal and parieto-occipital cerebral areas might also have impaired semantic fluency (26, 27). The fluctuation in cognition in patients with LBD might influence the difference in VFT results (28). Cognitive decline can persist for long periods in AD, whereas cognition might improve in LBD.

The strengths of the present study include the investigation of the resting-state characteristics of brain activity and VFT in patients with AD and LBD using fNIRS. Our findings revealed evidence of cognitive impairment and functional deficits in the prefrontal, local parietal, and temporal cortices under both conditions and suggests that fNIRS could serve as a reliable and valuable clinical diagnostic tool for differentiating LBD from AD. However, owing to the limited number of channels, the brain regions measured by fNIRS were restricted to the prefrontal and temporal cortex. Future studies with larger samples are needed. The interpretation of our findings should consider two key methodological constraints. First, the modest sample size may limit the statistical power to detect subtle between-group differences in functional connectivity, particularly in small brain regions. Second, our focus on prefrontal and parietal cortices (based on prior evidence of neurodegeneration in AD) precludes conclusions about other regions that may contribute to cognitive decline. Future studies with larger cohorts and whole-brain analyses are needed to validate these results and explore broader neural networks.

## 5 Conclusion

Non-invasive fNIRS indicators combined with blood biomarkers can differentially diagnose patients with AD and LBD. Levels of cognition-related blood biomarkers correlated with mean HbO concentrations while resting and during the VFT, suggesting their potential as indicators or biomarkers for different types of dementia.

## Data Availability

The original contributions presented in the study are included in the article/supplementary material, further inquiries can be directed to the corresponding authors.
